# Numerical simulations of the soliton dynamics for a nonlinear biological model: Modulation instability analysis

**DOI:** 10.1371/journal.pone.0281318

**Published:** 2023-02-16

**Authors:** Miguel Vivas-Cortez, Saima Arshed, Maasoomah Sadaf, Zahida Perveen, Ghazala Akram

**Affiliations:** 1 Escuela de Ciencias Físicas y Matemáticas, Facultad de Ciencias Exactas y Naturales, Pontificia Universidad Católica del Ecuador, Quito, Ecuador; 2 Department of Mathematics, University of the Punjab, Quaid-e-Azam Campus, Lahore, Pakistan; 3 Department of Mathematics, Lahore Garrison University, Lahore, Pakistan; China University of Mining and Technology, CHINA

## Abstract

This article deals with studying the dynamical behavior of the DNA model proposed by Peyrard and Bishop. The proposed model is investigated using the unified method (UM). Unified method successfully extracts solutions in the form of polynomial and rational functions. The solitary wave solutions and soliton solutions are constructed. An investigation of modulation instability is also presented in this paper. 3D and 2D plots are presented to exhibit the physical behavior of some of the obtained solutions.

## 1 Introduction

Nonlinear phenomena has become an interesting topic for research during last few decades. The nonlinear evolution equations are the key to examine and analyze problems found in numerous fields like biology, zoology, physics, chemistry, optics, fluid mechanics and geophysics. A variety of nonlinear models are investigated by different researchers like nonlinear Schrödinger equation [1–3], geophysical Korteweg-de Vries equation [4], Ablowitz-Kaup-Newell-Segur equation [5], (3 + 1)sine-Gordon equation [6] and many other equations. Various models are examined in detail for deriving soliton solutions [7–10].

In recent decades, soliton theory has become the popular topic of research and has motivated the researchers to develop new techniques for extracting soliton solutions of NLEEs. Recently, Li, Tian, Yang and Fan have done some interesting work in proving the soliton resolution of Wadati-Konno-Ichikawa equation and complex short pulse equation. They developed steepest descent method to study the long-time asymptotic behavior of the solutions of these equations, and gave a detailed proof of the soliton resolution conjecture and the asymptotic stability of solutions for these equations. Soliton resolution of N-soliton solution for the Wadati-Konno-Ichikawa equation is discussed in [11]. The asymptotic stability is also discussed. Soliton resolution for the afore-mentioned is studied in [12] for weighted Sobolev initial data. The complex short pulse equation is explored for soliton resolution using steepest descent method [13]. A number of novel and effective techniques have been reported for the soliton solutions in recent years. Riemann-Hilbert problem is constructed in [14] for solving nonlinear Schrödinger equation. Hirota bilinear method is used to study the (2+1)-dimensional Sawada-Kotera equation [15]. Soliton solutions are extracted for the three-component coupled Hirota equations using $\bar{\partial }$-dressing method [16]. Ansatz method, sub-equation method and explicit power series method [17] are employed on (2+1)-dimensional nonlinear Schrödinger equation. The freak wave solutions of the nonlinear Schrödinger equation are determined using Darboux transformation combined with separation of variable method [18]. Two exact solution methods are applied on Lakshmanan-Porsezian-Daniel model to extract soliton solutions in [19, 20]. The exp-function method is applied to solve some nonlinear partial differential equations (NLPDEs) [21]. Nonlinear Schrödinger equation is explored by using modified Khater method [22].

The main idea of this paper is to employ, the unified method, on the proposed model. As a result, few new soliton solutions have been reported for the first time in this paper. The unified scheme has solutions of two types: polynomial function solutions and rational function solutions. These two types of solutions have been further divided as solitary wave solution, soliton rational solution, periodic rational solution, etc. The unified method has been successfully utilized in other problems as well and many interesting results are reported [23, 24].

The molecular form of DNA is twice helical, which indicates that it has side-by-side strands that are twisted around each other. Dauxois developed the most polished form of Peyrard-Bishop model for DNA [25–27]. The Hamiltonian of the hydrogen links for the strings perforation is described in [28–31]. The mathematical form of the governing model is as follow:

$$\begin{array}{c} R_{tt}-\left(a_{1}+3a_{2}R_{x}^{2}\right)R_{xx}-2\eta \;\zeta e^{-\eta R}(e^{-\eta R}-1)=0, \end{array}$$ (1)

where *a*_1_ and *a*_2_ are the inter-site nucleotide distance in the DNA strands [29, 31].

The article is structured as follows: Section 3 presents the unified method to examine polynomial function and rational function solutions. Section 4 presents the application of the explained method on the aforementioned model of DNA. The instability analysis for the considered problem is presented in Section 5. Section 6 presents the graphical illustrations. The last section contains the concluding remarks.

## 2 Unified method [32]

The NLPDE is considered in the form

$$\begin{array}{c} P(R,R_{x},R_{t},R_{\text{xx}},R_{\text{xt}},R_{\text{tt}},\ldots )=0, \end{array}$$ (2)

where *R* = *R*(*x*, *t*). The suitable traveling wave transformation *R*(*x*, *t*) = *r*(*ξ*), *ξ* = *x* − *ωt* permits to change Eq (2) into ODE.

The algorithmic steps for finding the traveling wave solution for the transformed ODE in polynomial and rational form via UM scheme are described as follows:

### 2.1 Polynomial solutions

For the polynomial solution of converted ODE, the substitution is assumed, as

$$\begin{array}{ccc} r(\xi ) & = & \sum_{k=0}^{n}A_{k}\lambda^{k}\left(\xi \right),\\ {\left(\lambda^{\prime }\left(\xi \right)\right)}^{q} & = & \sum_{k=0}^{qn}B_{k}\lambda^{k}\left(\xi \right),\;\;q=1,2, \end{array}$$ (3)

where *A*_*k*_ and *B*_*k*_ are arbitrary constants. Polynomial soliton solutions are further classified into solitary wave solutions, soliton wave solutions and elliptic wave solutions depending on the value of *n* and *q*.

### 2.2 Rational solutions

For the rational solution of converted ODE, the substitution is assumed, as

$$\begin{array}{ccc} r(\xi ) & = & \frac{\sum_{k=0}^{n}A_{k}\lambda^{k}\left(\xi \right)}{\sum_{k=0}^{m}C_{k}\lambda^{k}\left(\xi \right)},\;\;n\geq m,\\ {\left(\lambda^{\prime }\left(\xi \right)\right)}^{q} & = & \sum_{k=0}^{qn}B_{k}\lambda^{k}\left(\xi \right),\;\;q=1,2, \end{array}$$ (4)

where *A*_*k*_, *C*_*k*_ and *B*_*k*_ are arbitrary constants. Like polynomial soliton solutions, the rational soliton solutions are also further classified as periodic rational solutions and soliton rational solutions.

## 3 The mathematical analysis

The exact solutions of Eq (1) has been extracted by considering the following transformation

$$\begin{array}{c} R(x,t)=r(\xi ),\;\;\;\xi =x-\omega t, \end{array}$$ (5)

where *ω* be the speed and *ξ* represents amplitude of traveling wave. Using the transformation Eq (5), the NLPDE is transformed into an ODE as,

$$\begin{array}{c} \omega^{2}\left(r^{\prime \prime }\right)-(a_{1}+3a_{2}\left(r^{\prime 2}\right)r^{\prime \prime }-2\eta \zeta e^{-\eta r}\left(e^{-\eta r}-1\right)=0. \end{array}$$ (6)

Multiplying Eq (6) by *r*′ and integrating, the following relation is obtained.

$$\begin{array}{c} \frac{\left(\omega^{2}-a_{1}\right)}{2}{\left(r^{\prime }\right)}^{2}-\frac{3}{4}a_{2}{\left(r^{\prime }\right)}^{4}+\zeta e^{-\eta r}\left(e^{-\eta r}-2\right)+C=0, \end{array}$$ (7)

where *C* being integration constant.

Substituting

$$\begin{array}{c} s\left(\xi \right)=e^{-\delta r(\xi )} \end{array}$$ (8)

into Eq (7), a nonlinear ODE is obtained as follow:

$$\begin{array}{c} \frac{\left(\omega^{2}-a_{1}\right)}{2\eta^{2}}s^{2}{\left(s^{\prime }\right)}^{2}-\frac{3}{4\eta^{4}}a_{2}{\left(s^{\prime }\right)}^{4}+\zeta s^{5}\left(s-2\right)+Cs^{4}=0. \end{array}$$ (9)

The homogenous balance principle between *s*′^4^ and *s*^6^ implies *n* = 2(*p* − 1), *p* = 2, 3…. For the sake of convenience, the values *p* = 2 and *q* = 1 or are taken.

### 3.1 Polynomial solutions

The Polynomial function solutions of Peyrard and Bishop model of DNA are as follow:

#### 3.1.1 Solitary wave solution

In order to get the solitary wave solution, taking *n* = 2 and *q* = 1 in Eq (3), the following relations are obtained.

$$\begin{array}{ccc} s(\xi ) & = & A_{0}+A_{1}\lambda \left(\xi \right)+A_{2}\lambda^{2}\left(\xi \right),\\ \lambda^{\prime }\left(\xi \right) & = & B_{0}+B_{1}\lambda \left(\xi \right)+B_{2}\lambda^{2}\left(\xi \right). \end{array}$$ (10)

Substituting Eq (10) in Eq (9) and equating the powers of *λ*(*ξ*) to zero, a system of algebraic equation has been obtained. The following solution is calculated.

$$\begin{array}{ccc} A_{0} & = & \mp \frac{2\sqrt{3}B_{0}B_{2}\sqrt{a_{2}}}{\eta^{2}\sqrt{-\zeta }},\;\;A_{1}\;\;=\;\;\mp \frac{2\sqrt{3}B_{1}B_{2}\sqrt{a_{2}}}{\eta^{2}\sqrt{-\zeta }},\;\;A_{2}\;\;=\;\;\mp \frac{2\sqrt{3}B_{2}^{2}\sqrt{a_{2}}}{\eta^{2}\sqrt{-\zeta }},\\ \omega & = & \mp \frac{\sqrt{\eta^{2}a_{1}-2\sqrt{-3\zeta a_{2}}\eta^{2}-3B_{1}^{2}a_{2}+12B_{0}B_{2}a_{2}}}{\eta }. \end{array}$$ (11)

Using Eqs (10) and (11), the solitary wave solution of Eq (1) is

$$\begin{array}{cc} R & =-\frac{1}{\eta }\text{ln}[\frac{\sqrt{3a_{2}}\left(B_{1}^{2}-4B_{0}B_{2}\right)}{\eta^{2}\sqrt{-\zeta }\left(1+\text{cos}\left[\left(\xi +c_{1}\right)\sqrt{\left(-B_{1}^{2}+4B_{0}B_{2}\right)}\right]\right)}]. \end{array}$$ (12)

Here, *c*_1_ is the integration constant, and *ξ* = *x* − *ωt* as defined in Eq (5).

#### 3.1.2 Soliton wave solution

In order to find optical soliton wave solution, taking *q* = 2 for Eq (3), the following relation is obtained.

$$\begin{array}{ccc} s(\xi ) & = & A_{0}+A_{1}\lambda \left(\xi \right)+A_{2}\lambda^{2}\left(\xi \right),\\ \lambda^{\prime }\left(\xi \right) & = & \lambda \left(\xi \right)\sqrt{B_{0}+B_{1}\lambda \left(\xi \right)+B_{2}\lambda^{2}\left(\xi \right)}. \end{array}$$ (13)

Substituting Eq (13) in Eq (9) and equating the powers of *λ*(*ξ*) to zero, a system of algebraic equation has been obtained. The following solution has been obtained.

$$\begin{array}{ccc} A_{0} & = & 0,\;\;A_{1}\;\;=\;\;\frac{\sqrt{3}B_{1}\sqrt{a_{2}}}{\eta^{2}\sqrt{-\zeta }},\;\;A_{2}\;\;=\;\;\frac{2\sqrt{3}B_{2}\sqrt{a_{2}}}{\eta^{2}\sqrt{-\zeta }},\\ B_{0} & = & \frac{1}{4}\frac{B_{1}^{2}}{B2},\;\;\omega \;\;=\;\;\frac{1}{2}\sqrt{\left(\frac{8\sqrt{-3\zeta a_{2}}B_{2}\eta^{2}-4\eta^{2}B_{2}a_{1}+3a_{2}B_{1}^{2}}{\eta^{2}B_{2}}\right)}B_{1}^{2}. \end{array}$$ (14)

Using Eqs (13) and (14), the soliton wave solution is retrieved, as

$$\begin{array}{c} R=-\frac{1}{2}\frac{\text{ln}3+2\text{ln}E}{\eta }. \end{array}$$ (15)

Here, $E_{1}=\frac{{B_{1}}^{3}}{B_{2}\eta^{2}}\frac{\sqrt{-\frac{p_{2}}{\xi }}e^{1/2\;(\xi +c_{1})\sqrt{\frac{{B_{1}}^{2}}{B_{2}}}}}{{\left(-2\;e^{1/2\;c_{1}\sqrt{\frac{{B_{1}}^{2}}{B_{2}}}}B_{1}+e^{1/2\;\xi \;\sqrt{\frac{{B_{1}}^{2}}{B_{2}}}}\right)}^{2}}$ or $E_{2}=\frac{{B_{1}}^{3}}{B_{2}\eta^{2}}\frac{\sqrt{-\frac{p_{2}}{\xi }}e^{1/2\;(c_{1}+\xi )\sqrt{\frac{{B_{1}}^{2}}{B_{2}}}}}{{\left(-2\;e^{1/2\;\xi \;\sqrt{\frac{{B_{1}}^{2}}{B_{2}}}}B_{1}+e^{1/2\;c_{1}\sqrt{\frac{{B_{1}}^{2}}{B_{2}}}}\right)}^{2}}$. *c*_1_ is the integration constant and *ξ* = *x* − *ωt* is defined in Eq (5).

#### 3.1.3 Elliptic wave solution

For the evaluation of elliptic wave solution, taking *n* = 2, *p* = 2 and *q* = 2, the following relations are obtained.

$$\begin{array}{ccc} s(\xi ) & = & A_{0}+A_{1}\lambda \left(\xi \right)+A_{2}\lambda^{2}\left(\xi \right),\\ \lambda^{\prime }\left(\xi \right) & = & \sqrt{B_{0}+B_{2}\lambda \left(\xi \right)+B_{4}\lambda^{4}\left(\xi \right)}. \end{array}$$ (16)

Substituting Eq (16) in Eq (9) and equating the powers of *λ*(*ξ*) to zero, a system of algebraic equation has been obtained. The following solution is obtained by the aid of Mathematica.

$$\begin{array}{ccc} A_{0} & = & \mp \frac{\sqrt{3}B_{2}\sqrt{a_{2}}}{\eta^{2}\sqrt{-\zeta }},\;\;A_{1}\;\;=\;\;0,\;\;A_{2}\;\;=\;\;\mp \frac{2\sqrt{3}B_{4}\sqrt{a_{2}}}{\eta^{2}\sqrt{-\zeta }},\\ \omega & = & \mp \frac{\sqrt{-\eta^{2}a_{1}-2\sqrt{-3\zeta a_{2}}\eta^{2}-6B_{1}^{2}a_{2}}}{\eta },\;\;B_{0}\;\;=\;\;\frac{B_{2}^{2}}{4B_{4}}. \end{array}$$ (17)

By Eqs (16) and (17), the solution of elliptic type has been obtained, as

$$\begin{array}{c} R=-\frac{1}{2}\frac{\left(\text{ln}\left(3\right)+2\text{ln}(-\frac{B_{2}p_{2}({\left(\text{JacobiSN}(\frac{1}{2}\sqrt{2}\sqrt{-B_{2}}\zeta +c_{1},\text{csgn}({B_{2}}^{2}))\right)}^{2}-1)}{\sqrt{-p_{2}\zeta }\eta^{2}})\right)}{\eta }, \end{array}$$ (18)

where *JacobiSN* represents the presences of Jacobi elliptic function which have different values with respect to different relation between the value of *B*_*i*_; *i* = 0, 2, 4…. Here, *c*_1_ is the integration constant and *ξ* = *x* − *ωt* is defined in Eq (5).

### 3.2 Rational function solution

Rational function solution for the above mentioned model is expressed in Eq (4). The homogenous balance principle for Eq (9) implies *m* − *l* = 2(*n* − 1) with *n* = 1, 2, 3, …. In order to get the solutions, *n* = 1(*m* = 1) and *q* = 2 are taken. The rational function solution are of two types; **periodic rational solution** and **soliton rational solution**.

#### 3.2.1 Periodic rational solution

The following relations are obtained for periodic rational solution.

$$\begin{array}{ccc} s(\xi ) & = & \frac{A_{0}+A_{1}\lambda \left(\xi \right)}{C_{0}+C_{1}\lambda \left(\xi \right)},\\ \lambda^{\prime }\left(\xi \right) & = & \sqrt{B_{0}^{2}-B_{2}^{2}\lambda^{2}\left(\xi \right)}. \end{array}$$ (19)

Substituting Eq (19) in Eq (9) and and equating the powers of *λ*(*ξ*) to zero, a system of algebraic equation has been obtained. The following solution is obtained.

$$\begin{array}{ccc} A_{0} & = & \mp \frac{\sqrt{3a_{2}}C_{1}B_{0}B_{2}}{\eta^{2}\sqrt{-\zeta }},\;\;A_{1}\;\;=\;\;0,\;\;C_{0}\;\;=\;\;\mp \frac{C_{1}B_{0}}{B_{2}},\;\;C_{1}\;\;=\;\;C_{1},\\ c & = & \frac{\eta^{2}a_{1}+3B_{2}^{2}a_{2}+2\eta^{2}\sqrt{-3\zeta a_{2}}}{\eta },\;\;A\;\;=\;\;\frac{1}{4}B_{2}^{2}\frac{3B_{2}^{2}a_{2}+4\eta^{2}\sqrt{-3\zeta a_{2}}}{\eta^{4}}. \end{array}$$ (20)

Using Eqs (19) and (20), the periodic rational solution is obtained, as

$$\begin{array}{c} R=-\frac{1}{2}\frac{\left(\text{ln}(3)+2\;\text{ln}(\frac{{B_{2}}^{2}\sqrt{-\zeta p_{2}}\text{csgn}(\frac{1}{\left(\text{cos}(B_{2}(c_{1}-\xi ))\right)})}{\eta^{2}\zeta \;(-\text{csgn}(B_{2})\text{sin}(B_{2}(c_{1}-\xi ))+\text{csgn}(\frac{1}{\left(\text{cos}(B_{2}(c_{1}-\xi ))\right)}))})\right)}{\eta }. \end{array}$$ (21)

Here, *c*_1_ is the integration constant, *B*_*i*_’s and *C*_*i*_’s are arbitrary constant and *ξ* = *x* − *ωt* as defined in Eq (5).

#### 3.2.2 Soliton rational solution

For soliton rational solution,

$$\begin{array}{ccc} s(\xi ) & = & \frac{A_{0}+A_{1}\lambda \left(\xi \right)}{C_{0}+C_{1}\lambda \left(\xi \right)},\\ \lambda^{\prime }\left(\xi \right) & = & \sqrt{B_{0}+B_{1}\lambda \left(\xi \right)+B_{2}^{2}\lambda^{2}\left(\xi \right)}. \end{array}$$ (22)

Substituting Eq (22) in Eq (9) and equating the power of *λ*(*ξ*) to zero, a system of algebraic equation has been obtained. The following solution is obtained by the aid of Maple.

$$\begin{array}{ccc} A_{0} & = & -\frac{1}{2}\frac{\sqrt{3a_{2}}C_{1}\sqrt{-4B_{0}B_{2}+B_{1}^{2}}}{\eta^{2}\sqrt{-\zeta }},\;\;A_{1}\;\;=\;\;0,\;\;C_{0}\;\;=\;\;\frac{1}{2}\frac{C_{1}(B_{1}+\sqrt{-4B_{0}B_{2}+B_{1}^{2}})}{B_{2}},\\ C_{1} & = & C_{1},\;\;\omega \;\;=\;\;\frac{\eta^{2}a_{1}-3B_{2}a_{2}+2\eta^{2}\sqrt{-3\zeta a_{2}}}{\eta }. \end{array}$$ (23)

Using Eqs (22) and (23), the solution is obtained, as

$$\begin{array}{c} R=-\frac{1}{2\eta }(4\text{ln}(2)+\text{ln}(3)+2\text{ln}\left(E\right)), \end{array}$$ (24)

where $E=(-\frac{\sqrt{p_{2}}{B_{2}}^{\frac{3}{2}}e^{(c_{1}+\xi )\sqrt{B_{2}}}}{\eta^{2}\sqrt{-\zeta }4\;e^{(c_{1}+\xi )\sqrt{B_{2}}}\sqrt{B_{2}}+\sqrt{\left(-4\;B_{0}B_{2}+{B_{1}}^{2}\right)}e^{2\;c_{1}\sqrt{B_{2}}}+4\;B_{2}e^{2\;\xi \;\sqrt{B_{2}}}})$, *c*_1_ is the integration constant and *ξ* = *x* − *ωt* is given as defined in Eq (5).

## 4 Modulation instability (MI) analysis

Linear stability analysis (LSA) is the cornerstone of modulation instability (MI) analysis. An investigation of modulation stability [3] of the DNA Peyrard-Bishop model has been conducted in this paper. A perturbed steady-state solution of the model is assumed, as

$$\begin{array}{c} R\left(x,t\right)=[\sqrt{Z_{0}}+\Gamma \left(x,t\right)]e^{ipZ_{0}x}, \end{array}$$ (25)

where incident power is represented by *Z*_0_ and Γ(*x*, *t*) is denoting the perturbation term. Substituting Eq (25) into Eq (1), the following result is obtained.

$$\begin{array}{c} \Gamma_{tt}+a_{1}\Gamma_{xx}+2\eta^{2}\zeta (\sqrt{Z_{0}}+\Gamma )=0. \end{array}$$ (26)

Here Γ* is the complex conjugate of Γ. To perform the instability analysis, the solutions are determined with the exponential growth, as

$$\begin{array}{c} \Gamma \left(x,t\right)=p_{1}e^{i(\kappa x-\Omega t)}+p_{2}e^{-i(\kappa x-\Omega t)}, \end{array}$$ (27)

where Ω is indicating the frequency, *κ* is expressing the wave number, whereas *p*_1_ and *p*_2_ are arbitrary constants. The following dispersion relation *κ* = *κ*(Ω) is obtained upon solving the homogenous system which is obtained by substituting the Eq (27) into Eq (26).

$$\begin{array}{c} \kappa =\pm \sqrt{\frac{-\Omega^{2}-2\left(1+\sqrt{Z_{0}}\right)\zeta \eta^{2}}{a_{1}}}. \end{array}$$ (28)

The investigation of linear stability of steady-state is evaluated using Eq (28). There are two conditions on *κ* *i.e.* if *κ* is real then the solution is stable and it is instable if *κ* is imaginary. In case, the solution is not real, there will be an exponential growth in the perturbation. Thus, for the existence of MI, the necessary condition is that

$$\begin{array}{c} \frac{-\Omega^{2}-2\left(1+\sqrt{Z_{0}}\right)\zeta \eta^{2}}{a_{1}}<0. \end{array}$$ (29)

The modulation instability gain spectrum is evaluated, as

$$\begin{array}{ccc} h(\Omega ) & = & 2Im(\kappa )\\ & = & 2(\frac{\Omega^{2}+2\left(1+\sqrt{Z_{0}}\right)\zeta \eta^{2}}{a_{1}}). \end{array}$$ (30)

**Fig 1:**
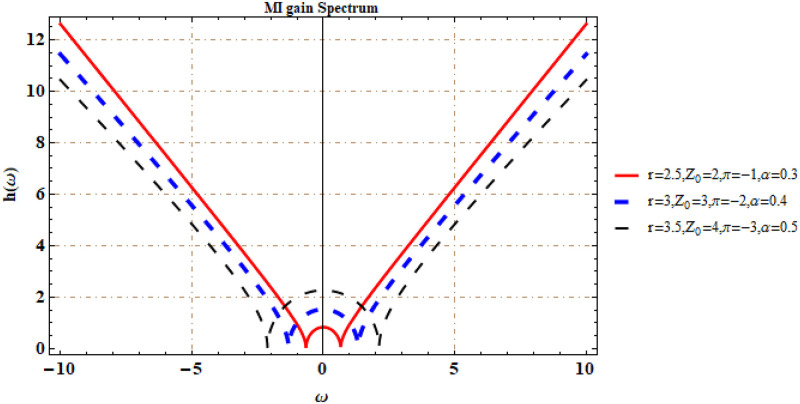
MI gain spectrum.

It is worth mentioning here that the dependency of modulation instability is on incident power *Z*_0_. It is illustrated in Fig 1 that an increase/decrease in the values of *Z*_0_ causes a decrease in the MI growth rate.

## 5 Graphical representation

**Fig 2:**
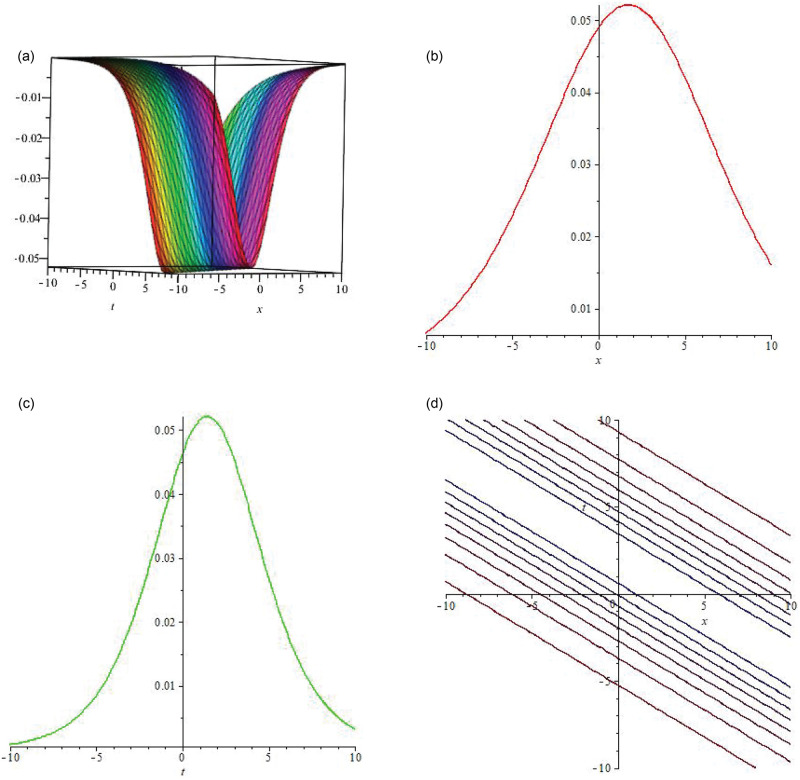
(a) is **3D solitary wave graph** of |*R*(*x*, *t*)|, (b) is **2D line graph** with the variation in x, (c) is **2D line graph** with the variation in t, (d) is the 2D contour plot.

**Fig 3:**
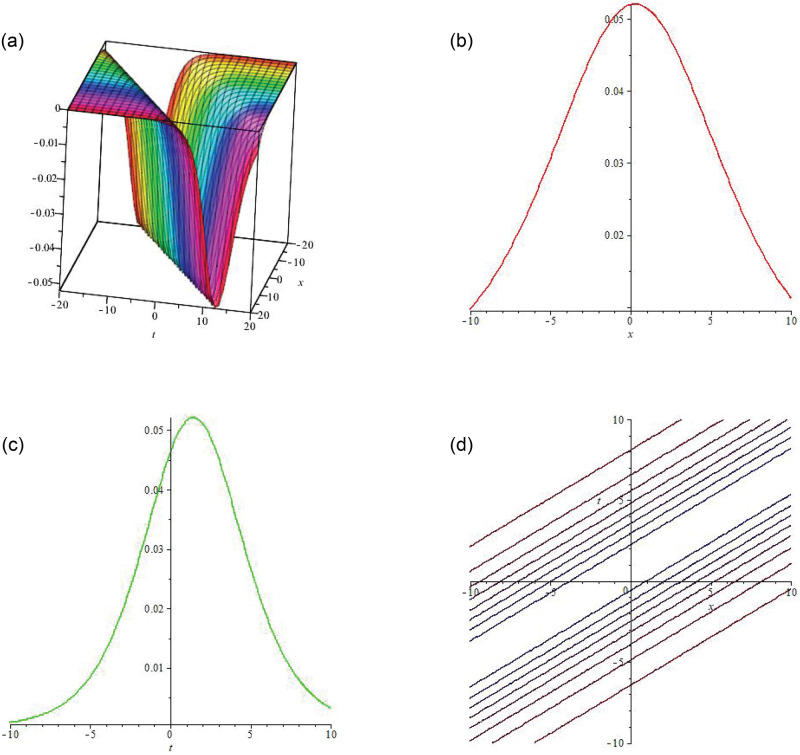
(a) is **3D solitary wave graph** of |*R*(*x*, *t*)|, (b) is **2D line graph** with the variation in x, (c) is **2D line graph** with the variation in t, (d) is the 2D contour plot.

**Fig 4:**
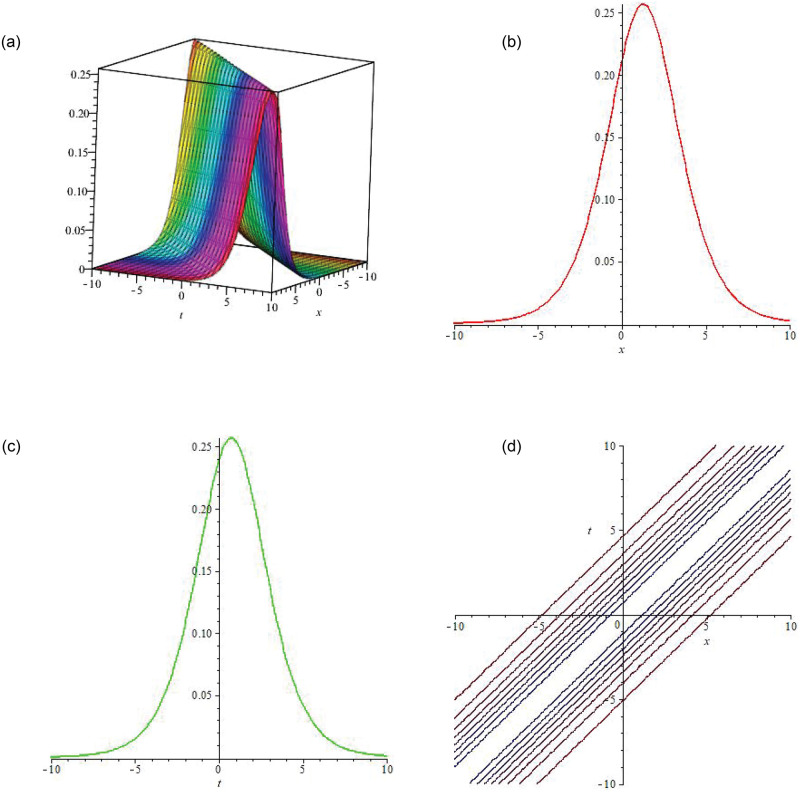
(a) is **3D soliton wave graph** of |*R*(*x*, *t*)|, (b) is **2D line graph** with the variation in x, (c) is **2D line graph** with the variation in t, (d) is the 2D contour plot.

Some of the solutions obtained by applying unified scheme on DNA Peyrard-Bishop model are graphically illustrated in this section. Figs 2–4 illustrate the solutions using 3-dimensional and 2-dimensional plots. In Fig 2 the soliton wave solution of the proposed model has been plotted. *R*(*x*, *t*) given by Eq (15) has been plotted by taking $B_{1}=-1,\;B_{2}=3,\;a_{1}=3,\;a_{2}=2.65,\;\eta =\frac{76}{9}$ and *ξ* = −0.001. Here the value of integration constant is *c*_1_ = 1 together with *E*_1_. In Fig 3, the soliton wave solution of the proposed model has been plotted. *R*(*x*, *t*) expressed by Eq (15) has been plotted by taking $B_{1}=-1,\;B_{2}=3,\;a_{1}=3,\;a_{2}=2.65,\;\eta =\frac{76}{9}$ and *ξ* = −0.001. Here the value of integration constant is *c*_1_ = 1 together with *E*_2_. Figs 2 and 3 are dark soliton wave solutions. In Fig 4, the soliton rational wave solution of the proposed model has been plotted. *R*(*x*, *t*) given by Eq (24) has been plotted by taking $B_{1}=-2.5,\;C_{0}=0.6,\;C_{1}=2,\;C_{2}=0.5,\;a_{1}=1,\;a_{2}=0.65,\;\eta =\frac{59}{9}$ and *ξ* = −0.001. Here, the value of integration constant is *c*_1_ = 0. The graphs in Fig 4 represent the bright soliton solution.

## 6 Concluding remarks

This paper reports solitons, solitary waves, elliptic waves and periodic rational solutions for a DNA Peyrard-Bishop model by employing a reliable and efficient analytical approach: the unified method. The modulation instability analysis of the considered model is also presented. The graphical analysis of few of the constructed results have been carried by 3D plots and 2D line plots upon choosing the specific values of arbitrary parameters.
